# Thermal proteome profiling: unbiased assessment of protein state through heat-induced stability changes

**DOI:** 10.1186/s12953-017-0122-4

**Published:** 2017-06-24

**Authors:** André Mateus, Tomi A. Määttä, Mikhail M. Savitski

**Affiliations:** European Molecular Biology Laboratory, Genome Biology Unit, Meyerhofstr. 1, 69117 Heidelberg, Germany

**Keywords:** Thermal proteome profiling, Protein thermal stability, Drug discovery, Target deconvolution, Mass spectrometry-based proteomics, Tandem mass tags

## Abstract

In recent years, phenotypic-based screens have become increasingly popular in drug discovery. A major challenge of this approach is that it does not provide information about the mechanism of action of the hits. This has led to the development of multiple strategies for target deconvolution. Thermal proteome profiling (TPP) allows for an unbiased search of drug targets and can be applied in living cells without requiring compound labeling. TPP is based on the principle that proteins become more resistant to heat-induced unfolding when complexed with a ligand, e.g., the hit compound from a phenotypic screen. The melting proteome is also sensitive to other intracellular events, such as levels of metabolites, post-translational modifications and protein-protein interactions. In this review, we describe the principles of this approach, review the method and its developments, and discuss its current and future applications. While proteomics has generally focused on measuring relative protein concentrations, TPP provides a novel approach to gather complementary information on protein stability not present in expression datasets. Therefore, this strategy has great potential not only for drug discovery, but also for answering fundamental biological questions.

## Background

Current drug discovery generally starts by using a target- or a phenotypic-based approach for compound screening [1]. In the former, a particular protein with an altered function or expression in a disease is targeted. This approach facilitates compound optimization, since structure-activity relationships are generally easy to establish [2]. However, disease-specific proteins are difficult to find and not all of them are ‘druggable’—the currently approved drugs only target around 900 proteins (including around 200 pathogen proteins) [3]. Further, the cellular context (i.e., subcellular location, post-translational modifications, levels of metabolites, and interactions with other proteins) is important for the function of proteins and is lost when working with purified proteins. These limitations have led, in recent years, to an renewed interest in phenotypic screening [4–6]. In this strategy, a particular trait or phenotype is sought in live cells (e.g., induction of cell death in cancer cells). The major challenge of phenotypic screening is the deconvolution of the mechanism of action of the putative drug molecules discovered during the screen. For that reason, multiple new methodologies for target identification have sprouted and have been extensively reviewed [7, 8].

A number of strategies use mass spectrometry-based proteomics [9, 10] and are based on changes in target stability upon compound binding. These include, for example, drug affinity responsive target stability (DARTS) [11], stability of proteins from rates of oxidation (SPROX) [12–14], or thermal proteome profiling (TPP) [15–18]. DARTS is based on limited proteolysis (LiP) [19], in which a low concentration of a protease with broad specificity is used to cleave only exposed regions of a protein (generally, loops or unfolded regions). DARTS exploits the fact that ligand binding can protect some of these regions from proteolysis [11]. In SPROX, aliquots of proteins are subjected to an increasing concentration of a chemical denaturant followed by oxidation of methionines that become exposed after unfolding [12–14]. Binding of a ligand stabilizes proteins against chemical denaturation. TPP exploits the differential stability of proteins after heat stress, i.e., proteins become more resistant to heat-induced unfolding when complexed with a ligand [15–18]. TPP can be applied in live cells, does not require compound labeling, and allows for an unbiased search of drug targets—to date, the only approach that combines all of these advantages. This review focuses on the principles of TPP, the recent advances in the method, and its possible future applications.

### From thermal shift assays to thermal proteome profiling

**Fig. 1:**
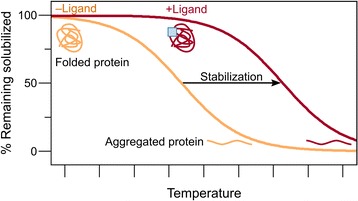
Principle of thermal shift assays. Proteins can be thermally stabilized by the presence of a ligand, leading to a higher apparent melting temperature (T_m_)

When proteins are subjected to a thermal stress, they generally irreversibly unfold, expose their hydrophobic core and subsequently aggregate (Fig. 1) [20, 21]. The temperature at which unfolding happens (the apparent melting temperature, T_m_) can be increased by the presence of a ligand, since part of the energy provided to the protein-ligand system is then used to dissociate the ligand from the protein (Fig. 1) [22–24]. This stabilization has been explored in purified proteins in structural biology [25, 26] and in drug discovery [27, 28]. The realization that this stabilization could be achieved directly in a cellular context [21] led to the development of the cellular thermal shift assay (CETSA) [29, 30]. For the first time, CETSA allowed the study of target engagement in cells and tissues. An advantage of using live cells is the possibility of monitoring the mechanisms of import and activation of pro-drugs. For example, methotrexate seems to be activated through polyglutamation prior to engagement of its target proteins dihydrofolate reductase (DHFR) and thymidylate synthase (TS)—inhibition of polyglutamate synthetase strongly decreased the stabilization of these targets by methotrexate [29]. While the initial CETSA protocol was based on immunoblot detection, microtiter-based formats have since been developed [30, 31]. This allowed the screen of intracellular target engagement of thousands of compounds and led to the discovery of a promising novel inhibitor of thymidylate synthase, CBK115334 [31]. However, since CETSA is based on an antibody readout, it is limited to the study of only a small number of proteins simultaneously. To allow proteome-wide studies of drug-protein interactions in a single experiment, the CETSA principles were combined with mass spectrometry-based proteomics [9, 32] in the thermal proteome profiling (TPP) approach [15–18]. This approach allows the unbiased search of direct targets and off-targets of drugs, as well as their indirect downstream effects on biochemical pathways (as discussed later).

### Thermal proteome profiling method

**Fig. 2:**
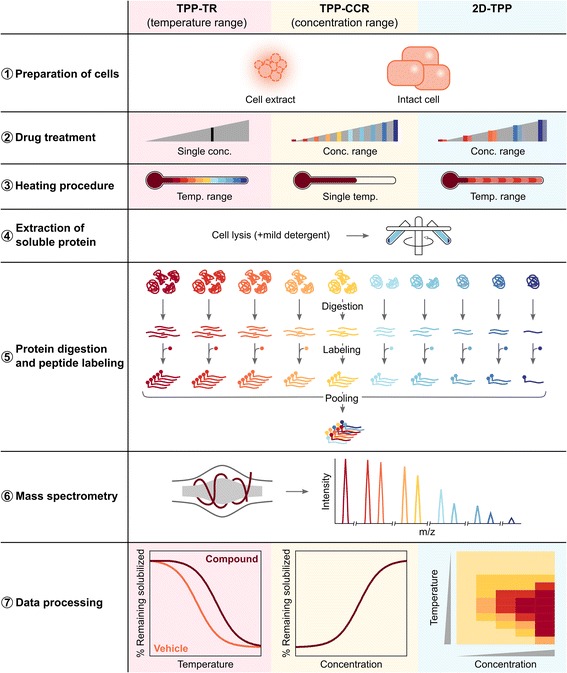
Thermal proteome profiling (TPP) method can be performed in one of three modes: temperature range (TPP-TR); compound concentration range (TPP-CCR); or two-dimensional TPP (2D-TPP). The general procedure is composed of (1) preparation of cells for the experiment, in which either cell extracts are prepared or intact cells are cultured; (2) drug treatment with either a single compound concentration (TPP-TR) or a range of compound concentrations (TPP-CCR and 2D-TPP); (3) heating the cells to a range of temperatures (TPP-TR and 2D-TPP) or a single temperature (TPP-CCR); (4) extraction of soluble protein fraction using ultracentrifugation after cell lysis—a mild detergent can be included to solubilize membrane proteins; (5) protein digestion using a proteolytic enzyme followed by peptide labeling with neutron-encoded isobaric tags (at this step, the illustration shows an example of the procedure for a TPP-TR experiment, but an analogous labeling scheme is used for TPP-CCR or 2D-TPP—see details in the main text); (6) mass spectrometric analysis using an Orbitrap mass spectrometer to resolve the 6 mDa differences between some of the adjacent TMT reporter ions (again, at this step, the illustration shows an example of the resulting spectra of one peptide following a TPP-TR experiment); and (7) data processing to obtain plots like the ones illustrated: for TPP-TR, melting curves for each protein in the absence of presence of drug will be generated—target engagement is observed as a shift in the apparent melting temperature (T_m_) of the protein; for TPP-CCR, potency curves for each protein will be obtained—from these curves it is possible to estimate the potency of the drug against each of the targets; for 2D-TPP, heat maps colored by the intensity of the abundance of soluble protein at each concentration and temperature will be generated

Since its original publication [15], the TPP method has been modified and expanded to tackle different challenges [15–18, 33–36]. However, the general outline of the procedure remains similar and can be described as: (1) preparation of cells for the experiment, (2) drug treatment, (3) heating procedure, (4) extraction of soluble protein fraction, (5) protein digestion and peptide labeling with tandem mass tags [37, 38], (6) mass spectrometric analysis, and (7) data processing (Fig. 2). These steps are described in more detail in the following sections.

### Preparation of the cells for the experiment: cell extracts vs. intact cells to distinguish direct and indirect targets

TPP can be performed on cell extracts, intact cells, or tissues. By lysing the cells prior to TPP, proteins, metabolites and co-factors are diluted. This should largely stop the normal metabolism of the cell and, therefore, stabilization of proteins will only be caused by the drug treatment (in other words, only direct targets will be identified). Conversely, in intact cell experiments, the cellular machinery is active and it is possible to observe not only the stabilization of the proteins to which the drug binds directly, but also the stabilization of downstream proteins resulting from the (in)activation or conformational change of the direct target. For example, in cell extracts, TH1579 only stabilized 7,8-dihydro-8-oxoguanine triphosphatase (MTH1), its known target [35]. However, in intact cells, the same compound also stabilized deoxycytidine kinase (dCK), an enzyme that recycles deoxynucleosides from degraded DNA—by catalyzing the phosphorylation of deoxycytidine, deoxyguanosine and deoxyadenosine [39]. Since MTH1 inhibition promotes DNA damage [40], this suggests that the pool of deoxynucleosides is increased and contributes to dCK stabilization.

### Drug treatment and heating procedure: single drug concentration vs. concentration range

After their preparation, the cells are incubated with the drug. At this step, either a single compound concentration is compared with a control, or a range of compound concentrations is applied. Generally, this choice is directly linked with the next step of the protocol—the heating procedure.

When a single compound concentration is used, the cells are heated up to a range of temperatures, and this experiment is termed temperature range TPP (TPP-TR). With this approach, it is possible to identify the majority of the targets of a compound, e.g., 49 of the 66 kinases that staurosporine inhibits, and that could be detected in K562 cell extracts, showed a reproducible shift in T_m_ of more than 1 °C [15]. However, there was a poor correlation between the magnitude of the T_m_ shift and the affinity of staurosporine to each kinase. This is because the extent of thermal stabilization depends not only on the affinity of the ligand, but also on the melting thermodynamics of the native protein.

To obtain affinity estimates with TPP, a compound concentration range TPP (TPP-CCR) can be performed. In TPP-CCR, cells are incubated with a range of concentrations of compound and heated to a single temperature. For example, K562 cell extracts incubated with a range of concentrations of GSK3182571 and heated to 53 °C showed a good agreement between the affinity determined in TPP-CCR and in kinobeads competition-binding experiments [15].

Recently, Becher et al. [18] developed a two-dimensional TPP (2D-TPP), in which cells are incubated with a range of compound concentrations and heated to multiple temperatures. This expansion allows an immediate estimate of compound affinity to the target and is much more sensitive at identifying targets. In one example, phenylalanine hydroxylase (PAH) was identified as an off-target of the histone deacetylase (HDAC) inhibitor panobinostat [18], which had not been possible with TPP-TR [16]. The reason for the substantial gain in sensitivity is that untreated and treated conditions are compared in the same mass spectrometry experiment, which yields more precise quantification (contrary to when two distinct experiments are compared, as is the case with TPP-TR). Further, in the 2D-TPP approach, the protein is expected to be stabilized in a dose-dependent manner, which adds an additional quality requirement to the data and filters out false positives [18].

### Extraction of soluble protein fraction

Following the heat treatment, the cells are lysed and proteins that have denaturated and aggregated are removed using ultracentrifugation. In the original protocol [15], membrane proteins were not analyzed, since all insoluble proteins were removed at this step. However, follow-up studies have shown that mild detergents can be used to include these proteins in the analysis without affecting heat-induced aggregation or promoting resolubilization of precipitated proteins [17, 33]. For example, the use of NP40 detergent did not affect the T_m_ values of proteins in Jurkat cells [17]. However, it allowed the identification of membrane proteins, such as tyrosine phosphatase CD45 (PTPRC) as well as other proteins of the T cell receptor (TCR) pathway, as the targets of pervanadate.

### Protein digestion and peptide labeling with isobaric tags

Once the soluble proteins are collected, they are digested using a general proteomics workflow (e.g., in-gel digestion [15–18], or in-solution digestion [33, 35]). The resulting peptides from each condition are then labeled using isobaric tandem mass tags (TMT) [41] and combined into a single sample to be analyzed by mass spectrometry. These tags, which when intact have the same mass, can be fragmented and yield reporter ions of different masses. This enables a quantitative comparison of multiple experimental conditions in the same mass spectrometry run. The recent expansion of TMT based quantification at first to eight [37, 42] and subsequently to ten conditions [38] was instrumental for the successful implementation of TPP. Particularly, in a TPP-TR experiment, the peptides from each temperature are labelled with a unique label, which allows the simultaneous quantification of the amount of soluble proteins at the different temperature conditions. This was key for the throughput and precision of the experiments. In a TPP-CCR experiment, each concentration condition is instead labelled with a unique label. To reduce the analysis time of a 2D-TPP experiment, while still having a reasonable resolution for compound concentration, five concentrations of compound are used at each temperature level [18]. In this way, peptides from each concentration of two adjacent temperatures are labelled with a unique tag from the same TMT10 set.

### Mass spectrometric analysis and data processing

So far only Orbitrap instrumentation [43] permits the analysis of neutron-encoded TMT10 tags, due to their capability to properly resolve the 6 mDa differences between some of the proximate TMT reporter ions. Following mass spectrometric analysis, protein identification and quantification is performed. For this purpose, a Python package (isobarQuant [44]) has been developed to be used together with the Mascot search engine (from Matrix Science [45]). While isobarQuant was specifically developed to address isobaric mass tag based quantification, other analysis platforms can also be used for this step, such as MaxQuant [46, 47] or ProteomeDiscoverer (Thermo Scientific). The analysis of the protein quantification data is then performed with the Bioconductor [48] TPP package [49], which also allows the analysis of 2D-TPP data. This package includes a statistical analysis step that highlights all the significant targets of a treatment.

### Future perspectives

**Fig. 3:**
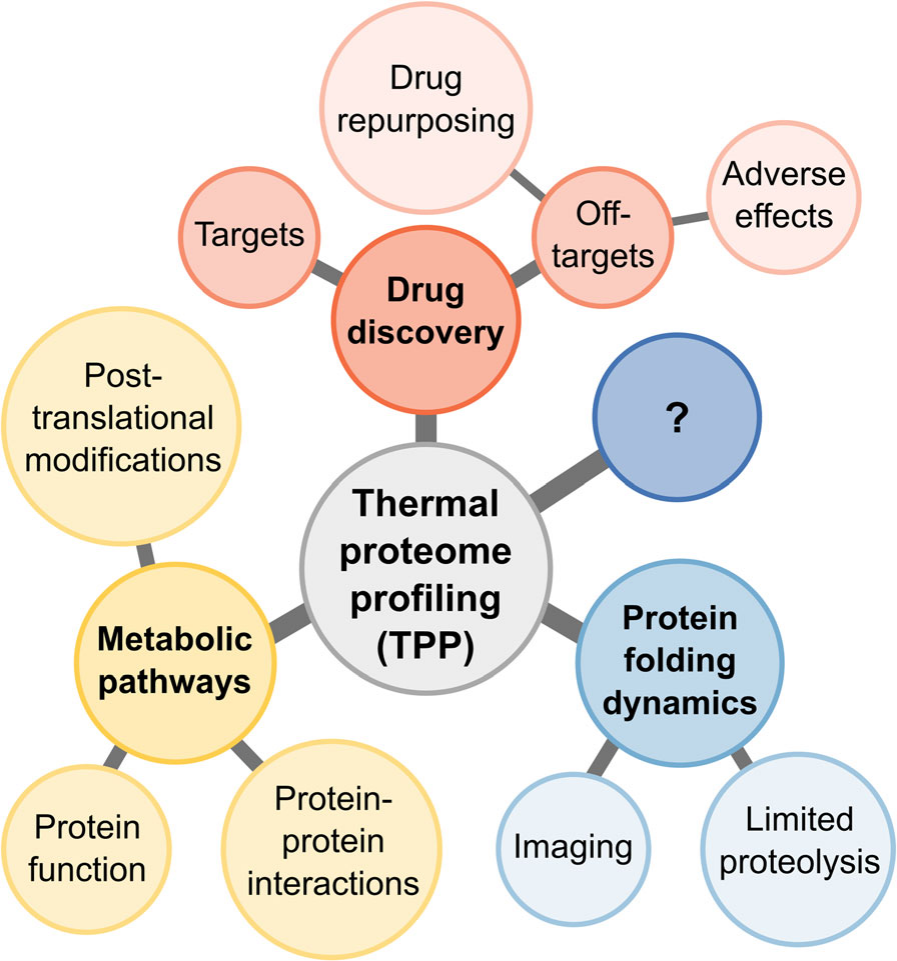
Examples of applications of thermal proteome profiling (TPP). In drug discovery, TPP can identify targets and off-targets. The latter can explain adverse effects or allow drug repurposing. TPP can also be used to explore metabolic pathways by studying post-translational modifications, protein-protein interactions or basic protein function. Further, TPP can be combined with limited proteolysis or imaging to provide further insight into protein folding dynamics. By being positioned at the border of proteomics and metabolomics, TPP can be applied to study many other biological questions

TPP was initially developed for the identification of compound targets and off-targets. In this regard, it was introduced as a new strategy to discover novel drug targets (for instance that brusatol is an inhibitor of global protein synthesis [34]), and off-targets that explain some of the adverse effects (e.g., alectinib and vemurafenib bind ferrochelatase (FECH), suggesting why they induce photosensitivity [15]). In addition, some of the new off-targets could potentially be used for drug repurposing (as an example, panobinostat binds and inhibits PAH, which might be used in tyrosinemia [18]) (Fig. 3). In the future, this approach could be pushed further, for example to find new antibiotic targets. Target identification by ligand stabilization (TILS), a technique based on similar principles to TPP, has recently shown that this method can be applied to bacteria and is not restricted to mammalian cells [50]. TILS relies on the analysis of the precipitate, rather than the remaining soluble fraction, and uses dimethyl labeling for protein quantification.

In principle, the thermal stability of any protein is affected by ligand binding to some extent. However, some target proteins show no statistically significant shifts in apparent melting temperature. For example, dasatinib did not show stabilization of its known target, BCR-ABL, despite the appearance of downstream target-related effects [15]. Also, some very low abundant proteins will not be identified by mass spectrometry, hence their stability will not be measured. Further, changes in stability of multi-domain proteins, for which only one domain is involved in ligand binding, will depend on the impact of the change in the whole protein (since protein denaturation and aggregation happens for the full-length polypeptide). Future improvement in instrumentation and sample preparation will lead to increased proteome coverage and enable identification of more low abundant targets (including cell surface proteins [51]), while newer TPP formats (2D-TPP) will help to identify even small stability shifts (as was seen with panobinostat and PAH stabilization [18]).

Besides drug discovery, TPP might become an important tool to map metabolic pathways, since it allows the study of post-translational modifications, protein-protein interactions, and the basic function of proteins (Fig. 3). For the study of post-translational modifications, it has been shown that phosphorylation affects protein thermal stability (e.g., pervanadate is known to induce phosphorylation of desmoglein-2 [52], a protein that was stabilized after treatment with this compound [17]). Protein-protein interactions can also be detected with TPP, since stability changes in proteins present in a complex can be identified (for example, kinase complexes containing cyclins were stabilized by the kinase inhibitor staurosporine [15]). To evaluate the function of a protein, the thermal profile of the proteome of cells in which the gene has been knocked-out might offer insight into the mechanism of the protein. The results from TPP could add an interesting layer to knock-out studies, since it is possible to see not only which proteins are stabilized, but also which proteins are destabilized. Destabilization can occur when a protein complex is disturbed or when the concentration of a metabolite is lowered (e.g., panobinostat lowers cholesterol levels and this contributes to a destabilization of apolipoprotein B [16]).

Further, TPP could be combined with other methods that complement protein thermal stability approaches (Fig. 3). For example, a recent study has profiled the melting proteome, in a lysate setting, using limited proteolysis, contributing insight into thermal unfolding at the sequence level [53]. Further understanding of folding and unfolding dynamics might be attained by a combination of single-molecule imaging and thermal stress.

## Conclusion

TPP is a recently developed tool that allows for the study of perturbations on thermal stability of the proteome. This provides information that is complementary to protein expression, since it is influenced by levels of metabolites, post-translational modifications and protein-protein interactions. TPP has been extensively used for the study of drug targets and off-targets.[19–22, 37–39] However, since this method lies at the interface between proteomics and metabolomics, it has a broad application and can be used to study many fundamental biological questions.

## Abbreviations

2D-TPP: Two-dimensional TPP
CETSA: Cellular thermal shift assay
DARTS: Drug affinity responsive target stability
LiP: Limited proteolysis
SPROX: Stability of proteins from rates of oxidation
TILS: Target identification by ligand stabilization
T_m_: Apparent melting temperature
TMT: Isobaric tandem mass tags
TPP: Thermal proteome profiling
TPP-CCR: Compound concentration range TPP
TPP-TR: Temperature range TPP
